# Impact of Endofungal Bacteria on Infection Biology, Food Safety, and Drug Development

**DOI:** 10.1371/journal.ppat.1002096

**Published:** 2011-06-30

**Authors:** Gerald Lackner, Christian Hertweck

**Affiliations:** Leibniz Institute for Natural Product Research and Infection Biology, HKI, Jena, Germany, and Friedrich Schiller University, Jena, Germany; University of California San Francisco, United States of America

The filamentous mould *Rhizopus microsporus* is a member of the zygomycetes (lower fungi). While some strains serve as food fermenting fungi, others represent infamous plant pathogens and opportunistic human pathogens. Recently, it was shown that some strains of *R. microsporus* are associated with symbiotic bacteria. Here, we outline why these organisms are important for human health and how they can be exploited for drug development. Furthermore, we illustrate what the investigation of bacterial–fungal symbiosis can teach us about the evolution of pathogenicity factors in general.

## *Rhizopus microsporus* Harbors Bacterial Endosymbionts as Intracellular Toxin Factories

**Figure 1:**
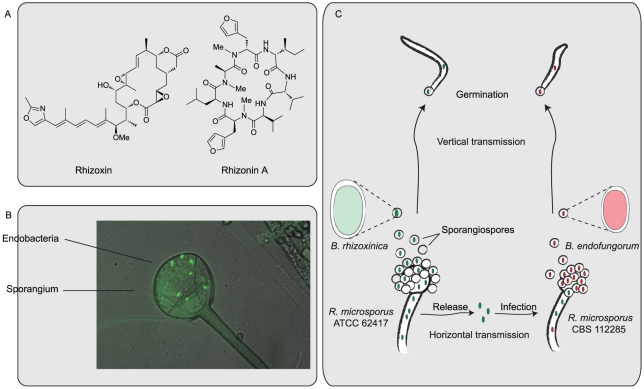
Chemical and biological features of endofungal bacteria. (A) Chemical structures of rhizoxin, an antimitotic macrolide, and rhizonin A, a hepatotoxic cyclopeptide. (B) Light micrograph of a sporangium of *R. microsporus* (ATCC 62147) stained with a viability assay system (Invitrogen). Green spots are living cells of the bacterial endosymbiont *B. rhizoxinica*. (C) Life cycle of *R. microsporus* strains and their endosymbionts *B. rhizoxinica* and *B. endofungorum*. Endobacteria are propagated within fungal spores (vertical transmission). Under laboratory conditions, isolated bacteria can infect compatible host strains (horizontal transmission).

*R. microsporus* (ATCC 62417) attacks rice plants and illicits rice seedling blight, a severe crop disease affecting rice fields in Asia [1]. Symptoms of the infection include abnormal swelling of the roots and finally death of the affected tissue. Plant pathogenic *R. microsporus* strains live as necrotrophic pathogens, i.e., they derive energy from killed host cells. For that purpose they secrete rhizoxin (see Figure 1A), a toxin that blocks mitosis in eukarytotic cells by binding to *β*-tubulin [2]. *Rhizopus* itself is resistant to the toxin due to an amino acid exchange in the tubulin protein [3]. The production of toxins by plant pathogenic fungi is a widespread virulence mechanism [4]. In the case of *R. microsporus*, however, the search for biosynthetic genes coding for rhizoxin biosynthesis led to an unexpected discovery: it is not the fungus itself that produces the pathogenicity factor rhizoxin. Instead, the fungus harbors bacterial symbionts, which are the actual producers of the virulence factor [1]. Toxin formation by bacteria has been demonstrated in analogy with Koch's postulates in classical microbiology: it was discovered that rhizoxin-producing strains of *R. microsporus* contained symbionts, while nontoxinogenic strains did not. Removal of symbionts by antibiotics indeed abolished rhizoxin production. The bacteria could be isolated and grown in axenic (pure) culture. Finally, reintroduction into cured hosts clearly reestablished rhizoxin production [1]. Intriguingly, microscopic investigations revealed that the bacteria are true endosymbionts, i.e., they inhabit the intracellular space of fungal mycelium (see Figure 1B). The first endosymbiont of *R. microsporus* that could be isolated was named *Burkholderia rhizoxinica* for its capability to produce rhizoxin [5].

## A New Role for Endobacteria in Mycotoxin Research and Food Safety

The discovery of endobacteria as producers of “mycotoxins” was relevant in the areas of natural product research and microbiology, since similar symbionts in other antibiotic producing fungi might have been overlooked in the past [6]. Indeed, another dangerous “mycotoxin”, rhizonin (see Figure 1C), originally isolated from *R. microsporus* (CBS 112285) is produced by endobacteria as well [7]. These endobacteria are related to *B. rhizoxinica*, but represent another species, *Burkholderia endofungorum* [5]. Rhizonin is a cyclopeptide, which is highly toxic for mammals. Tested animals exhibited serious hepatic lesions and died from chronic failure of the liver [8]. The fact that the producing strain was found on ground nuts in Mozambique underlines the relevance of the *Burkholderia*–*Rhizopus* symbiosis for food safety and human health. Concerning food safety, it is even more distressing that another *Burkholderia*–*Rhizopus* association was isolated from a tempe/sufu starter culture in Vietnam [1]. Tempe and sufu are traditional soy preparations in Asia that are fermented with *R. microsporus*. It could be demonstrated that rhizoxin is indeed produced during sufu fermentation, thus revealing a potential threat to human health [9].

## Endosymbionts of *Rhizopus* Were Detected in Human Pathogens but Are Not Essential for Zygomycoses

*Rhizopus* species including *R. microsporus* are regularly involved in zygomycoses (mucormycoses), disastrous fungal infections that affect immunocompromised patients. These diseases have high mortality rates [10] and are hard to treat by antifungal agents. Consequently, surgical debridement of infected tissue is often necessary [11]. After the discovery of *B. rhizoxinica* it was speculated whether toxin production by endosymbionts might enhance the virulence of fungal strains involved in human disease. The bacteria would then provide a promising target for the treatment of *Rhizopus* infections. Indeed, it has been shown that rhizoxin-producing strains are frequently involved in zygomycosis [11].

However, several lines of evidence suggest that rhizoxin production seems not to be essential for *Rhizopus* pathogenicity [11], [12]. First of all, clinical cases of zygomycosis are frequently caused by non-toxinogenic *Rhizopus* species [12]. Furthermore, when *R. microsporus* was cured from symbionts with antibiotics, it still retained its ability to infect mice under laboratory conditions [11]. Although these results show that endosymbionts are not the key players in zygomycosis, they might still raise the potential health threat caused by *R. microsporus*: it is well conceivable that endosymbionts that are released from fungal mycelium might cause sepsis or further complications. In particular, *B. rhizoxinica* and *B. endofungorum* could cause potential long-term damage by secretion of toxins into the human body. In fact, related *Burkholderia* strains have been isolated from clinical specimen [13]. Consequently, the existence of toxinogenic endosymbionts should be kept in mind when it comes to treatment of zygomycosis.

## Investigation of Rhizoxin Biosynthesis Can Promote Antitumor Therapy

Despite all theses dangers emanating from toxin-producing *Rhizopus/Burkholderia* strains, rhizoxin could also assist in antitumor therapy: due to its ability to block mitosis via binding to *β*-tubulin, rhizoxin exhibits strong activity against tumor cell lines in vitro [14]. The substance has already tested in phase II clinical trials [15]. However, its in vivo activity was unsatisfactory, probably due to low activity and rapid elimination from plasma [15], and thus it would be desirable to produce more potent derivatives. Rhizoxin belongs to the family of macrolide antibiotics (as e.g., erythromycin) and is produced by a modular polyketide synthase. The enzymatic assembly line catalyzes repetitive condensation of activated acetate (acetyl-CoA and malonyl-CoA) units, in a similar way as in fatty acid biosynthesis [16]. In contrast to the latter, polyketide biosynthesis allows for variable degrees of chain processing, and the polyketide backbone is further modified by tailoring enzymes. Through genetic manipulation of the biosynthesis genes it is possible to engineer polyketide biosynthesis, and thus create new derivatives. Indeed, it was possible to identify the genes coding for rhizoxin biosynthesis [17] and to modify these genes in isolated symbionts. Thus, the function of some enzymes could be investigated in detail [18], [19]. Furthermore, it is promising that cultivation of *B. rhizoxinica* in pure culture could significantly increase the yield of rhizoxin production and lead to the isolation of new, significantly more active rhizoxin derivatives [20].

## Endosymbionts of *Rhizopus*: Former Pathogens of Pathogens?

**Figure 2:**
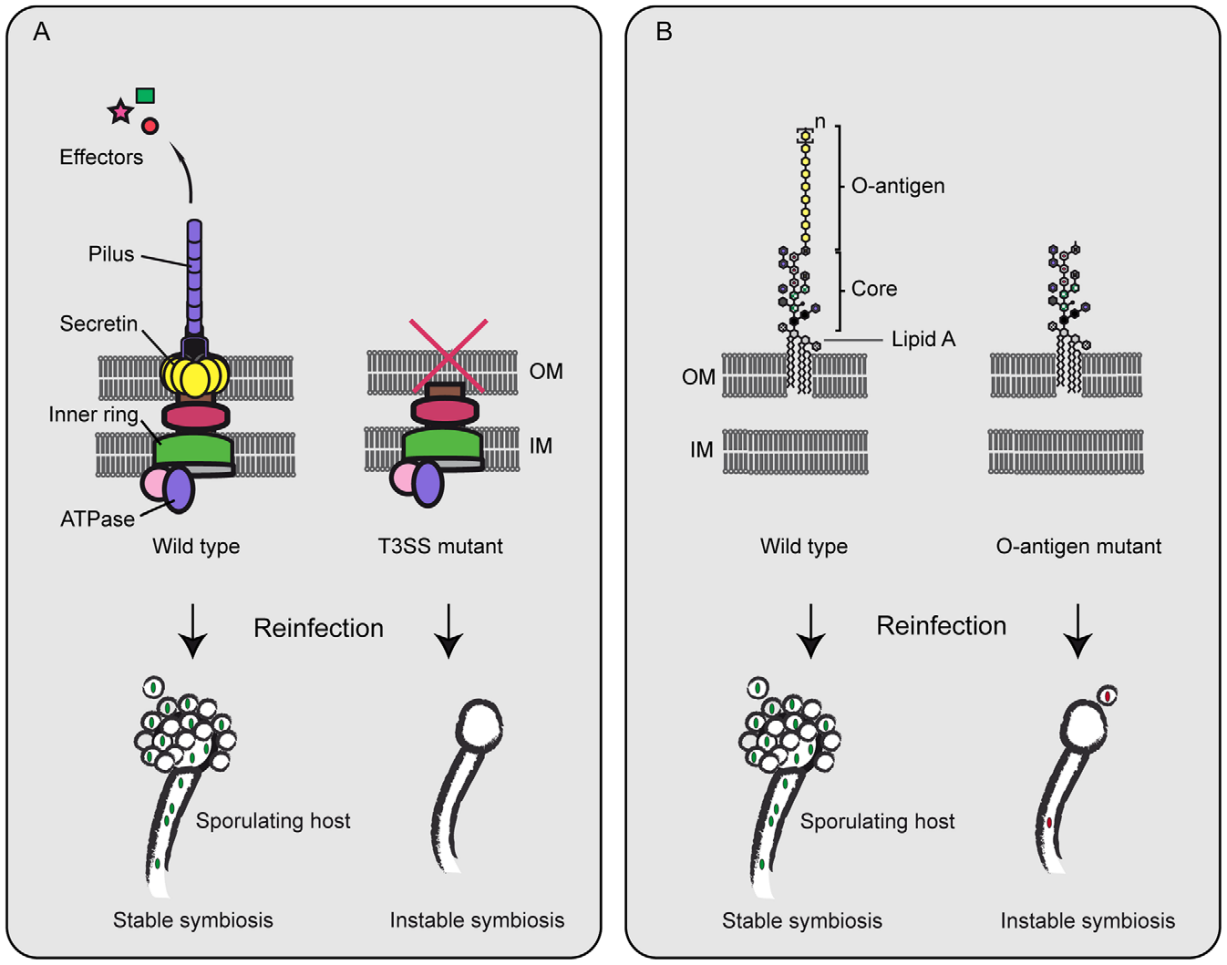
Symbiosis factors of *B. rhizoxinica*. (A) The role of the type III secretion system (T3SS) in bacterial–fungal symbiosis. T3SSs consist of several ring-like structures anchored in both the outer (OM) and inner membrane (IM) of Gram-negative bacteria. Energy is derived from an ATPase component situated at the cytosolic side of the protein complex. Effector proteins are secreted through a pilus structure into host cells. When key components are inactivated, mutants fail to reinfect the host. (B) Role of the lipopolysaccharide (LPS) in bacterial–fungal symbiosis. LPS molecules are anchored in the outer membrane of Gram-negative bacteria by their lipid component (lipid A). The sugar components form a heterogeneous core oligosaccharide and a polymeric O-antigen. When the O-antigen is missing, mutants reinfect the host sporadically, but are incapable of establishing a stable symbiosis.

From a biologist's point of view, the *Burkholderia*–*Rhizopus* symbiosis has some intriguing aspects that go beyond zygomycosis and natural product research. For instance, the question was raised as to how the association between *Rhizopus* and *Burkholderia* has evolved and how it is maintained. Microscopic investigations with GFP-labeled endobacteria revealed that endosymbionts enter fungal spores to be “inherited” during vegetative reproduction [21]. Intriguingly, the reproduction of the host has been hijacked by the symbionts: the host is unable to sporulate when endosymbionts are removed [21]. Thus, they ensure their own propagation alongside the host lineage (vertical transmission, see Figure 1C). In addition, endobacteria are able to infect compatible host organisms in laboratory cultures [22]. Spread of symbionts by release and infection of another host is called horizontal transmission (see Figure 1C). Both vertical and horizontal transmission could be shown to be relevant during evolution of the *Burkholderia*–*Rhizopus* alliance: comparison of phylogenetic trees of host strains and the corresponding symbiont strains revealed that the topologies of these trees resemble each other [23]. This means that host fungi and symbionts have undergone “cospeciation”—symbiont lineages have been associated with their fungal host lineage over a long period of time. However, some host switching events could be detected, which must be due to horizontal transmission. These results suggest that endosymbionts have actually evolved from former parasites (pathogens) of *Rhizopus*. It looks like both partners benefit from their close relationship: while the fungus obtains powerful chemical weapons produced by *B. rhizoxinica*, the latter is supplied with nutrients and a safe niche. Any mutually beneficial symbiosis is called mutualism. However, the line between mutualism and parasitism is thin and shifts might occur during evolution. This parasitism–mutualism shift hypothesis is further supported by genome sequencing of *B. rhizoxinica* [24], [25]: while the genome retains only a reduced number of genes compared to free-living *Burkholderia* species, it encodes a large repertoire of typical virulence factors. It could be demonstrated that a type III secretion system (T3SS) [22] and the lipopolysaccharide O-antigen [26] are essential to maintain the symbiosis. Both factors are known pathogenicity factors of animal and plant pathogens. T3SSs are giant protein export machineries anchored in both membranes of Gram-negative bacteria (see Figure 2A). Usually, they accomplish export of effector proteins that manipulate cellular processes of the host [27]. Symbionts lacking these systems are unable to reinfect their host fungus and cannot illicit its sporulation like the wild type [22]. Similar results were obtained for mutants lacking the O-antigen—a long polysaccharide chain anchored in the outer membrane (see Figure 2B) [26]. It is plausible that the O-antigen is needed for protection against fungal defense mechanisms or recognition of the symbionts.

These results teach us that bacterial symbionts of fungi employ similar factors as plant parasites or human pathogens. These mechanisms seem to be universal host control tools that can be adapted to highly versatile hosts and cellular targets.
